# Novel Variation in the External Carotid Artery: Implications for Clinical and Surgical Practice

**DOI:** 10.7759/cureus.66580

**Published:** 2024-08-10

**Authors:** Jonathan S Okereke, Vivian T Nguyen, Kristy M Pham, Claire R Perkins, Allie Peever, Adegbenro O Fakoya

**Affiliations:** 1 Anatomy, Louisiana State University Health Sciences Center, Shreveport, USA; 2 Cellular Biology and Anatomy, Louisiana State University Health Sciences Center, Shreveport, USA

**Keywords:** common trunk, cadaveric dissection, anatomical variation, ascending pharyngeal artery, external carotid artery

## Abstract

The external carotid artery (ECA) is a major branched artery that supplies head and neck structures. An undocumented variation of the ECA was discovered during cadaveric dissection of the anterolateral cervical region, in which a common origin for the ascending pharyngeal, facial, and lingual arteries was identified. In addition, bilateral, duplicate ascending pharyngeal arteries (APAs) were identified at the aforementioned common trunk and the bifurcation of the external and internal carotid arteries. Anatomical knowledge regarding the location of the APA is essential to physicians, as this vessel is a primary supply source for many skull base tumors and vascular lesions. Furthermore, such anatomical knowledge is essential to physicians, as there have been cases of misdiagnosis regarding APA anomalies as an internal carotid artery (ICA) dissection. In this cadaver, both ECAs exhibited typical branching into the superior thyroid artery (STA), occipital artery, posterior auricular artery, maxillary artery, and superficial temporal artery.

## Introduction

The external carotid artery (ECA) gives rise to numerous arterial branches, including the superior thyroid artery (STA), ascending pharyngeal artery (APA), lingual artery (LA), facial artery (FA), occipital artery, posterior auricular artery, maxillary artery, and superficial temporal artery. The ECA and its numerous branches serve as the major vascular supply source to the face and cervical region [1]. The specified arterial branches typically deviate at separate points as the ECA ascends from the common carotid artery (CC). However, it is possible to find anatomic variability regarding the branching patterns of these arteries. In a prior cadaveric study, Devadas et al. determined that the linguofacial common trunk was the highest occurring variant (20%) to the ECA [2]. Devadas et al. also estimated that accessory branches derived from the ECA at a 7.5% incidence and even identified a singular, unilateral thyrolinguofacial trunk among their cases [2].

In this report, two novel unilateral variations will be discussed in detail. Both variations were discovered during cadaveric dissection of the anterolateral cervical region. Our first variation was the presence of an anomalous common trunk that originated from the ECA and gave rise to the FA, LA, and one of two APAs. This discovery was particularly significant considering the limited literature detailing similar common trunks in other cadaveric dissections. Our second variation was the presence of bilateral, duplicate APAs in the anterolateral cervical region, which this case report highlights as a new and previously unreported anomaly. Both findings point towards the importance of correct patterning and knowledge of anatomical variants in arterial branching, as these foundational understandings are essential aspects of diagnostic processes and surgery [3].

## Case presentation

**Figure 1:**
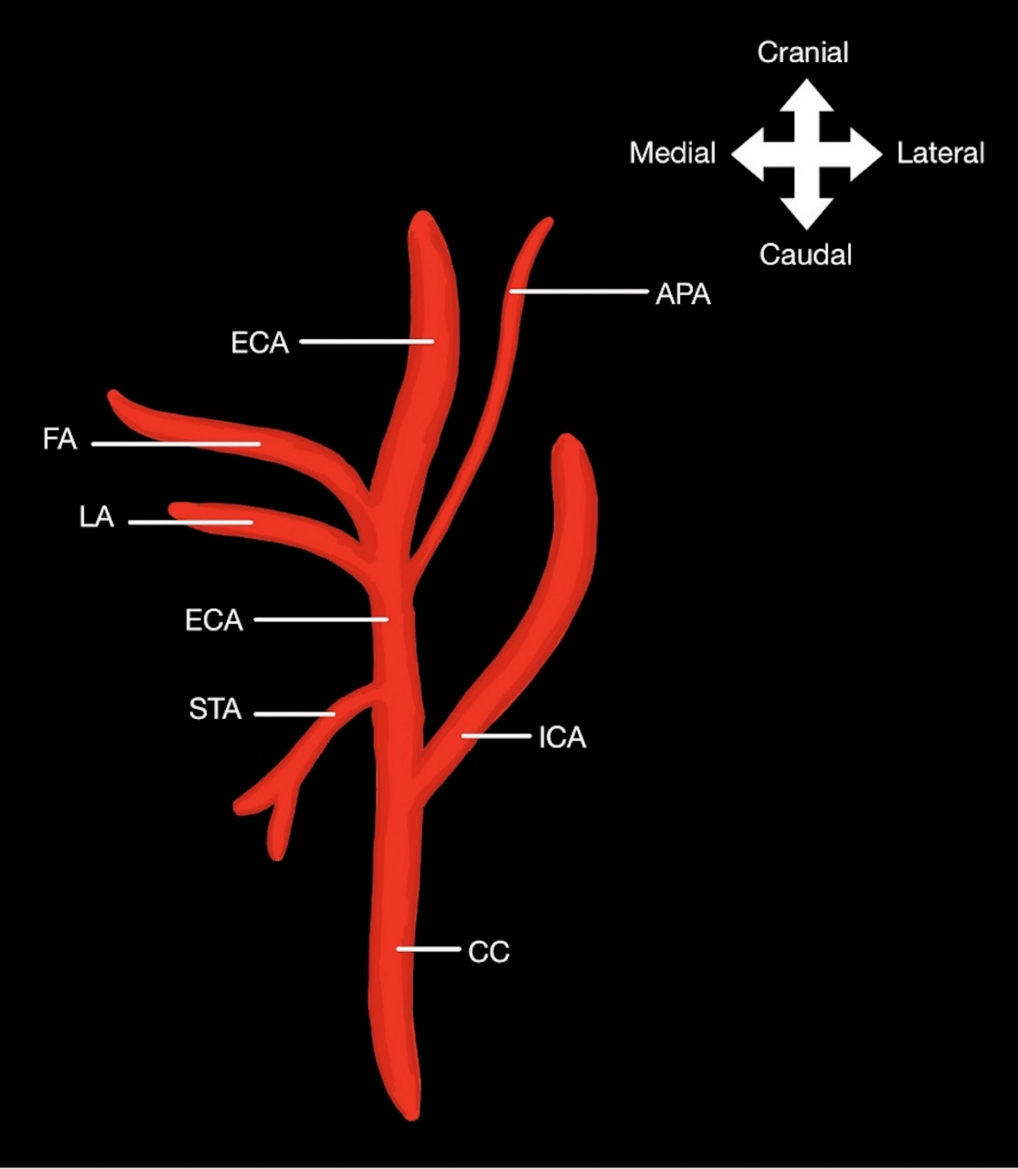
Diagram of the typical branching pattern of the lingual, facial, and ascending pharyngeal arterial branches of the external carotid artery ECA: External carotid artery; CC: common carotid artery; STA: superior thyroid artery; LA: lingual artery; FA: facial artery; APA: ascending pharyngeal artery; ICA: internal carotid artery This image has been created by one of the authors, Vivian T. Nguyen, using Notability software (Ginger Labs, Inc., San Francisco, CA)

During routine dissection of the anterolateral cervical region of an 85-year-old white female cadaver, an anomaly involving the ECA and its branches was discovered. Traditionally, the LA, FA, and APA arise from different segments of the ECA, as depicted in Figure 1 [1]. 

**Figure 2:**
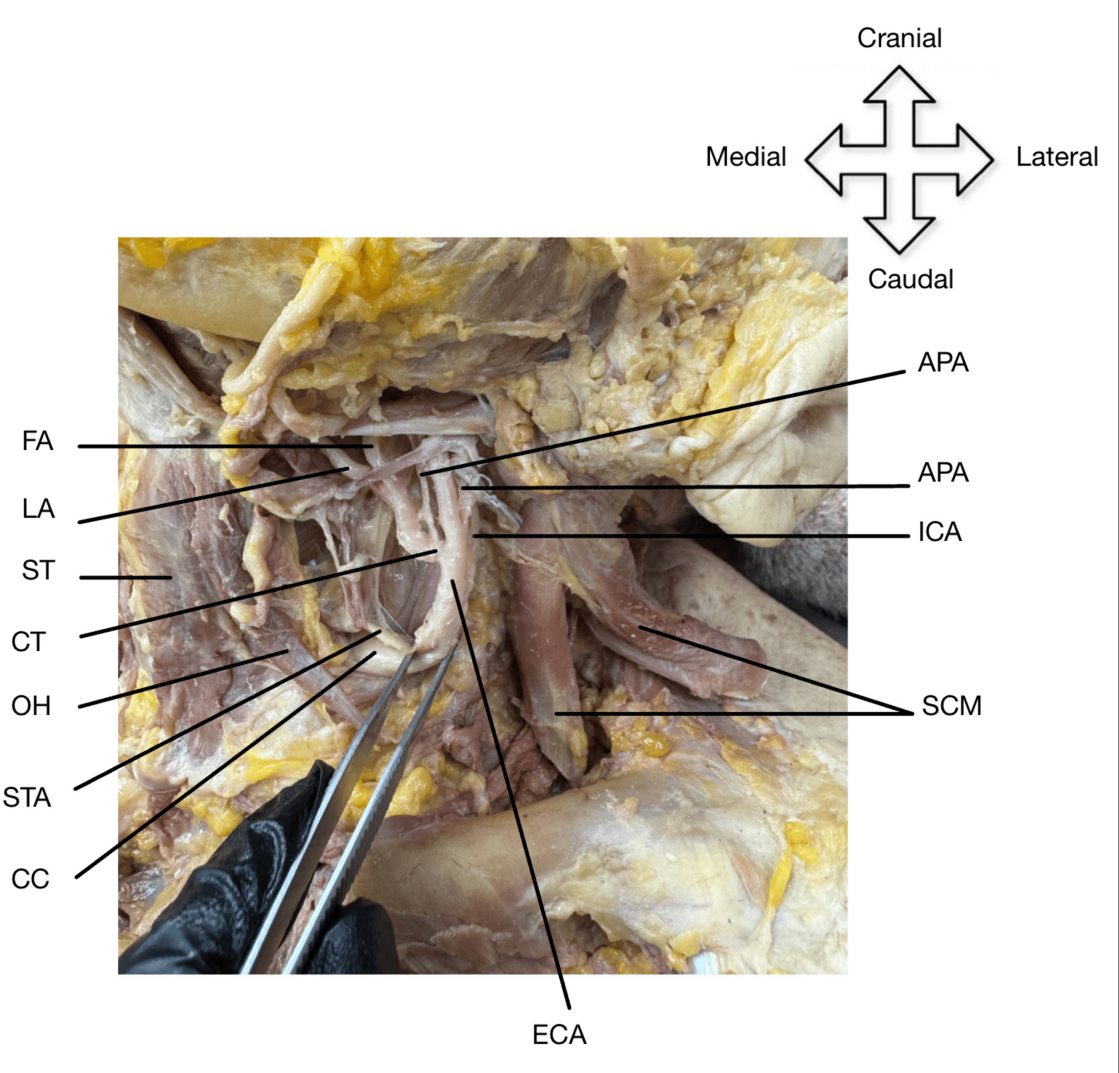
Variant branching of the ECA The ECA gives off the STA, which descends medially. The ECA also gives off the APA, which arises in between the bifurcation of the CC into the ECA and ICA. The ECA then gives off the LA and FA and a duplicate APA, which all ascend medially from a common trunk. ECA: external carotid artery; CC: common carotid artery; OH: omohyoid; STA: superior thyroid artery; CT: common trunk; ST: sternothyroid; LA: lingual artery; FA: facial artery; APA: ascending pharyngeal artery; ICA: internal carotid artery; SCM: sternocleidomastoid

**Figure 3:**
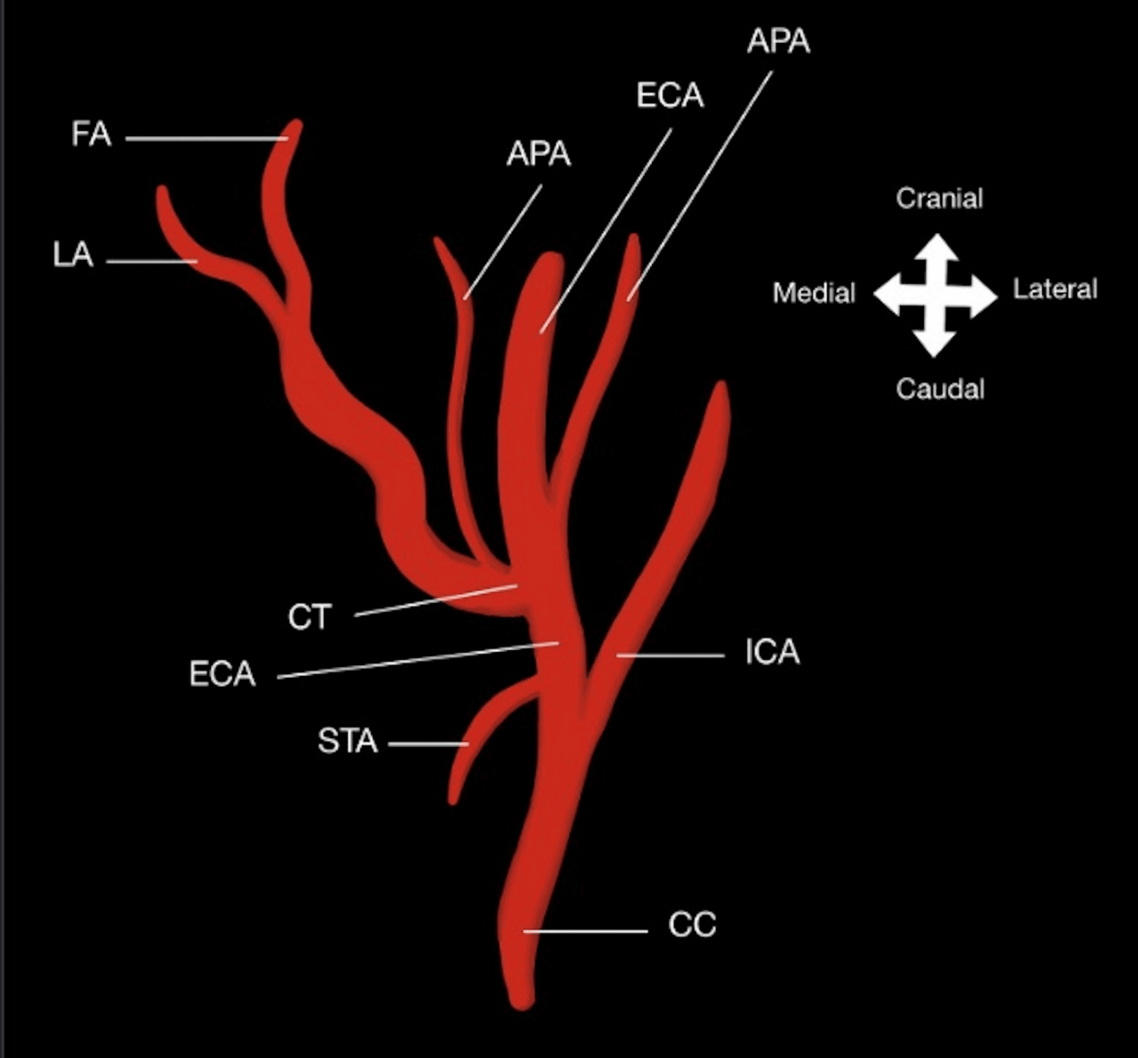
Diagram of variant branching of the ECA The ECA gives off the STA, which descends medially. The ECA also gives off the APA, which arises in between the bifurcation of the CC into the ECA and ICA. The ECA then gives off the LA and FA and a duplicate APA, which all ascend medially from a common trunk. ECA: external carotid artery; CC: common carotid artery; STA: superior thyroid artery; CT: common trunk; LA: lingual artery; FA: facial artery; APA: ascending pharyngeal artery; ICA: internal carotid artery This image has been created by one of the authors, Vivian T. Nguyen using Notability software (Ginger Labs, Inc., San Francisco, CA)

Following skin removal and superficial fascia removal, the sternocleidomastoid (SCM) was reflected to reveal the CC. The CC was followed superiorly until its bifurcation into the ECA and internal carotid artery (ICA). An interesting discovery was then made upon closer inspection of the ECA. The LA, FA, and APA all shared a common trunk that emerged from the ECA, as shown in Figure 2 and Figure 3.

Additionally, a duplicate APA branch was identified. The first APA originated laterally from the common trunk relative to the LA and FA, while the second APA originated between the bifurcation into the ECA and ICA, both shown in Figure 2 and Figure 3, respectively. This anomaly was not an isolated occurrence and was seen bilaterally in the right and left anterolateral cervical regions of our cadaver. 

## Discussion

Previous literature has identified common trunk variations for the facial and lingual arteries with varying incidence between 7% and 20%; however, to the best of our knowledge, there has not been another case describing a common origin for the ascending pharyngeal, facial, and lingual arteries until now [3]. Our case is also the first to uncover bilateral, duplicated APAs at the aforementioned common trunk and the bifurcation of the external and internal carotid arteries. Such a discovery has significant clinical implications, as the ascending pharyngeal artery is a primary source of skull base tumors and vascular lesions, including meningiomas, giant cell tumors, and arteriovenous fistulas or malformations [4]. Furthermore, Babic et al. report misdiagnosing an ICA dissection due to an APA variation, demonstrating the importance and relevance of embryonic development and aortic arch morphology [5].

While it is impossible to point towards erroneous vascular remodeling, excessive angiogenic factors in utero, or some other underlying cause as the sole perpetrator behind the two anomalies, as Tropius et al. recognize, vascular irregularities such as these may have profound and widespread ramifications [6]. Injury to this common trunk, either through trauma, tumor compression, infectious and pathogenic spread, or thrombosis, can involve large regions of the face, oral cavity, and pharynx, in addition to some cranial nerve aspects and other subsequent anastomoses [4, 7].

Surgical intervention is yet another common injurious agent that implicates vasculature and, in this case, could impact various regions of the head and neck through the shared common trunk. According to the American Academy of Otolaryngology-Head and Neck Surgery, per annum in the United States, over 500,000 tonsillectomy cases occur amongst children younger than 15 years, with facial artery hemorrhage estimated at an incidence between 3% and 3.9% amongst all procedures [8,9]. Moreover, the APA has been identified as a source of post-tonsillectomy hemorrhage after uvulopalatopharyngoplasty (UPPP), the most common surgical procedure to treat obstructive sleep apnea amongst the 17% and 34% of women and men, respectively, affected in the United States [10, 11]. With an ascending pharyngeal hemorrhage incidence ranging between 0.2% and 2.2% post-UPPP in the first 24 hours and then 0.1% to 4.8% afterward, together with the context of facial artery hemorrhages and tonsillectomies, while an effective treatment option, as alluded to before, surgical intervention does indeed risk injury and compromise to oral and maxillofacial vasculature [10].

In a similar yet separate vein, a traumatic lingual hematoma can cause tongue enlargement that compromises the upper airway, necessitating immediate endotracheal intubation; this risk is further exacerbated by patients with coagulopathy or on anticoagulant treatment [12]. Endotracheal intubation as a procedure, although rare, does carry tongue perforation and lingual hematoma risk [13,14]. As such, with an estimated 13 to 20 million intubations performed in the United States each year with varying expected difficulty and complication, and as anticoagulant use and insurance claims have risen from $25.9 to $33.8 million between 2014 and 2019, practicing physicians must proceed with caution while attempting intubation before surgical cases or during resuscitative efforts to reduce lingual artery injury and airway obstruction risk [15-17].

Therefore, as our colleagues articulated in a prior report, vascular anomalies continuously reinforce the importance of angiographic studies before surgical cases [18]. Because of the commonality and frequency of tonsillectomy, intubation, and anticoagulant use, these procedures and practices in the context of anatomical variation amongst the facial, lingual, and ascending pharyngeal arteries could result in vascular injury or disruption to previously mentioned structures and regions in unsuspecting individuals. Angiographic studies can then minimize incidental complications by elucidating the surgical area prior to incision or intubation, which is vital to mitigating intra- or postoperative complications and improving patient care [19].

## Conclusions

Exploring possible arterial supply variations is important for anatomical understanding and clinical practice. Although rare, acknowledging the existence of anatomical variations can significantly impact surgery and medical management. As previously discussed, abnormal branching of arteries can precipitate misdiagnoses and poor patient outcomes from surgery or other invasive procedures when proper evaluation is not performed. Therefore, it is crucial that these anatomical variations continue to be explored and presented.
